# Comparison of case-based learning and traditional lecture in teaching residents on research misconduct: a controlled before-and-after study

**DOI:** 10.1371/journal.pone.0322336

**Published:** 2025-04-25

**Authors:** Lulin Chen, Yizhao Li, Xiaoyan Guo, Wei Liu

**Affiliations:** 1 Department of Science and Education,The Second Nanning People’s Hospital, Nanning, People’s Republic of China; 2 School of Foreign Language, Guangxi University of Science and Technology, Liuzhou, People’s Republic of China; Institute of Medical Biochemistry Leopoldo de Meis (IBqM) - Federal University of Rio de Janeiro (UFRJ), BRAZIL

## Abstract

As scientific outputs continue to surge, research misconduct has garnered global attention. Case-based learning (CBL), an active student-centered learning strategy, possesses many advantages but has not been widely used in China due to resource constraints. This study aimed to address the research gap regarding the impact of CBL and traditional lecture on residents’ knowledge and attitudes towards research misconduct. This controlled before-and-after study was conducted at two tertiary hospitals in southwest China from November 2022 through March 2023. All medical residents at the two hospitals were defined as participants. Residents participating in CBL course at one hospital comprised the experimental group, whereas those engaging in traditional lecture at another hospital constituted the control group. The CBL and control group included 202 and 205 individuals, respectively. A total of 298 subjects were successfully matched after propensity score matching, with 149 individuals in each group. After the courses, the participants’ knowledge on research misconduct, perceived consequences for research misconduct, and their agreement rate regarding research misconduct improved in the CBL and control group (*P* < 0.05), but certain aspects of their perceived consequences and agreement rate did not show significant improvement in the control group. The results revealed that there is a marked enhancement in residents’ knowledge about research misconduct, their perception of its consequences, and their overall disapproval of such behavior in the CBL group. This underscores the effectiveness of CBL in fostering a deeper understanding and stronger aversion towards research misconduct among residents.

## Introduction

As the number of scientific outputs continues to proliferate, research misconduct is attracting increasing attention globally. In recent years, Chinese scientific output has surged significantly, accounting for 23.4% of the total scientific papers and 27.2% of the top 1% most highly cited papers between 2018 and 2020, surpassing the United States [1]. The significant increase in scientific outputs has also drawn international scrutiny towards research misconduct in China. According to a research report from Nature, Chinese first nationwide review of retractions and scientific misconduct has revealed over 17,000 retractions involving Chinese co-authors since 2021 [2]. The Retraction Watch Database reports that 5561 articles were retracted from China in 2023, representing 78.5% of the total retracted articles globally. Previous studies have demonstrated a relatively high prevalence of research misconduct among doctors, nurses, and residents in hospitals [3–5], underscoring a necessity for enhancing research integrity courses.

Case-based learning (CBL) is an active learning strategy that places students at the center of the learning process, emphasizing their centrality in the educational environment and foster a community-oriented exploration of realistic and specific situations [6]. CBL is results-driven compared with traditional lecture, emphasizing the development of students’ meticulous logical reasoning skills. Teachers pose questions in real cases, prompting students to synthesize their acquired knowledge to analyze, deduce, and ultimately resolve issues. This method is used in many medical fields and is considered to be more effective than traditional lecture [7,8].

Due to limitations in manpower, time, and location, CBL has not been fully promoted and implemented [9]. Traditional lecture is still the most frequently utilized instructional method in medical and clinical education in China [10] and is perceived as a teacher-centered educational approach, in which knowledge transmitted by and from the teacher and passively absorbed by the students [11]. These result in the ineffectiveness of traditional lecture in imparting crucial critical reasoning skills required in higher education, especially in specialized courses. To the best of our knowledge, there has been no study that examine the impact of CBL and traditional lecture on resident’ knowledge and attitudes towards research misconduct. Therefore, this study aimed to address these gaps in southwest China, seeking to gain a deeper understanding of the effects of these two teaching methods on residents’ comprehension and perspectives towards research misconduct.

## Materials and methods

### Study design

This controlled before-and-after study was conducted at two tertiary hospitals in southwest China from November 21, 2022 through March 12, 2023. The study had no exclusion criteria, and all medical residents at the two hospitals were defined as participants. Residents at one hospital participated in CBL course and were defined as the experimental group, while those at other hospital participated in traditional lecture and were defined as the control group. All CBL course and traditional lecture were designed and conducted by the same research investigator, and were limited to about 1.5 hours. Participants’ knowledge and attitudes towards research misconduct were compared before and after the courses by using an online questionnaire. The results of the training were evaluated by the other member of the research team to reduce bias. After reviewing the online informed consent form, which outlines the purpose, methodology, content of the study, participants can indicate their willingness to participate in the research by clicking “Agree” to proceed with the survey. Once the questionnaire was completed, it was returned along with the informed consent content. The residents could get the teaching subject of their lesson, but they were not informed that there were different formats.

### Instruments

The questionnaire consists of 4 parts, including demographic characteristics and research experience, knowledge on research integrity, perceived consequences for research misconduct and attitudes towards research misconduct, and it has been described in our previous study [5]. The responses to parts 2–3 were expressed by a 5-point Likert-type scale. Participants could answer part 4 using “agree”, “neutral” or “disagree”.

### Ethics approval

The study was approved by institutional review board of The Second Nanning People’ s Hospital (No.Y2022043).

### CBL course

The CBL course was designed according to a previously published conceptual framework for case-based learning [12]. The course was focused on 2 cases with each case taking half an hour. The first case was relatively straightforward and the second case was more complex so as to be educational and challenging for all participants. Participants were divided into small groups with those at different residency year evenly distributed among the groups. To enhance the engagement of participants in courses, a readiness assessment exercise consisting of 3 questions was conducted at the beginning of all cases. The detailed information of the case was presented in PowerPoint, and the residents were given 15 minutes to discuss the role behaviors, violations of regulations, and attitudes towards the role. Next, the research investigator would ask the 3 questions to each group, and summarize the discussions to ensure the progress of the course after completing all cases.

### Traditional lecture

The readiness assessment exercise was also conducted in this group. Residents were called upon to answer the same questions after the research investigator presented the same PowerPoint slideshow with the CBL group that detailed the typical cases of research misconduct and knowledge on research integrity.

### Statistical analysis

SPSS 25.0 (IBM, Chicago, IL, USA) was used to analyze the data. Frequencies and percentages are utilized to depict categorical variables. Mean and standard deviation are used to express normally distributed variables, and median and interquartile range (IQR) are used to express skewed variables. The total score was derived by summing the responses on the 5-point Likert scale. Statistical analyses including the Chi-squared or Fisher’s exact test for categorical variables, Student’s t test for normally distributed variables, and the Mann-Whitney U test for skewed variables were used to compare the differences in knowledge and attitude towards research misconduct before and after the courses. We used the unbalanced characteristics between the experimental group and the control group as matching variables, employed logistic regression to calculate the propensity score for each individual, utilized “nearest neighbor matching” to find individuals with similar propensity scores, and generated a matched dataset. A *P* value less than 0.05 was considered statistically significant.

## Results

Participants’ demographic characteristics are shown in Table 1. The CBL and control group included 202 and 205 individuals, respectively. There were statistically differences in residency year and educational status between the two groups (*P* < 0.05). To balance the significant confounding variables potentially affecting participants’ knowledge and attitude toward research misconduct, a matched population with a 1:1 ratio between the CBL and control group was established utilizing the propensity score matching (PSM) method after adjusting residency year and educational status. Ultimately, a total of 298 subjects were successfully matched, with 149 individuals in each group. This process effectively eliminated all significant imbalances, without yielding other imbalanced variables between the two groups.

**Table 1:** Distribution of characteristics between CBL group and control group.

Characteristics	Overall data				Propensity 1:1 Matching			
	CBL group (%) (n=202)	Control group (%) (n=205)	χ^2^	*P* ^a^	CBL group (%) (n=149)	Control group (%) (n=149)	χ^2^	*P* ^a^
Residency year								
1	55 (27.2)	91 (44.4)	31.594	<0.001	52 (34.9)	53 (35.6)	0.886	0.642
2	106 (52.5)	52 (25.4)			57 (38.3)	50 (33.6)		
3	41 (20.3)	62 (30.2)			40 (26.8)	46 (30.9)		
Educational status								
Undergraduate or below	182 (90.1)	196 (95.6)	4.669	0.031	141 (94.6)	141 (94.6)	<0.001	1.000
Postgraduate or above	20 (9.9)	9 (4.4)			8 (5.4)	8 (5.4)		
Serving as an investigator for a research project								
No	154 (95.6)	159 (77.6)	0.100	0.751	116 (77.9)	120 (80.5)	0.326	0.568
Yes	48 (23.8)	46 (22.4)			33 (22.1)	29 (19.5)		
Publishing papers as a co-authors								
No	173 (85.6)	180 (87.8)	0.413	0.520	128 (85.9)	130 (87.2)	0.116	0.734
Yes	29 (14.4)	25 (12.2)			21 (14.1)	19 (12.8)		
Attending a course on research integrity								
No	123 (60.9)	136 (66.3)	1.306	0.253	95 (63.8)	48 (32.2)	0.537	0.464
Yes	79 (39.1)	69 (33.7)			54 (36.2)	101 (67.8)		

^a^The comparison between the two groups was conducted using the chi-squared test.

Table 2 shows the comparison of knowledge on research integrity before and after the courses. The total score of knowledge on research integrity increased from 35.00 (25.00, 39.00) to 48.00 (47.00, 60.00) in the CBL group (*P* < 0.05), and those in the control group increased from 32.00 (24.00, 36.00) to 48.00 (41.50, 57.00) (*P* < 0.05). All the listed items showed significant score increases in both groups (*P* < 0.001). The concept of duplicate publication scored the highest and the documentation on scientific integrity issued by regulatory authorities scored the lowest after the courses in both groups.

**Table 2:** Comparison of knowledge on research integrity before and after the courses [Median (IQR)].

Items	CBL group^a^ (n=149)		Control group^a^ (n=149)	
	Pre-course	Post-course	Pre-course	Post-course
Documentation on scientific integrity issued by regulatory authorities	2.00(2.00,3.00)	4.00(3.00,5.00)***	2.00(2.00,3.00)	4.00(3.00,4.00)***
Definition of research Integrity	3.00(2.00,4.00)	4.00(4.00,5.00)***	3.00(2.00,3.00)	4.00(3.00,5.00)***
Definition of fabrication	3.00(2.00,4.00)	4.00(4.00,5.00)***	3.00(2.00,3.00)	4.00(3.00,5.00)***
Definition of falsification	3.00(2.00,4.00)	4.00(4.00,5.00)***	3.00(2.00,3.00)	4.00(3.00,5.00)***
Definition of plagiarism	3.00(2.00,4.00)	4.00(4.00,5.00)***	3.00(2.00,3.00)	4.00(4.00,5.00)***
Inappropriate authorship	3.00(2.00,3.50)	4.00(4.00,5.00)***	3.00(2.00,3.00)	4.00(3.50,5.00)***
Concept of multiple submission	3.00(2.00,4.00)	4.00(4.00,5.00)***	3.00(2.00,3.00)	4.00(4.00,5.00)***
Concept of duplicate publication	3.00(2.00,4.00)	4.00(4.00,5.00)***	3.00(2.00,3.00)	4.00(4.00,5.00)***
Citation rules	3.00(2.00,3.00)	4.00(4.00,5.00)***	3.00(2.00,3.00)	4.00(3.00,5.00)***
Research ethics	3.00(2.00,3.00)	4.00(4.00,5.00)***	3.00(2.00,3.00)	4.00(3.00,5.00)***
Use of Research Funding	3.00(2.00,3.00)	4.00(4.00,5.00)***	2.00(2.00,3.00)	4.00(3.00,5.00)***
Disposition of research misconduct	3.00(2.00,3.00)	4.00(4.00,5.00)***	2.00(2.00,3.00)	4.00(3.50,5.00)***
Total score	35.00(25.00,39.00)	48.00(47.00,60.00)***	32.00(24.00,36.00)	48.00(41.50,57.00)***

^***^ *P* < 0.001

^a^The comparison before and after the course was conducted using the Mann-Whitney U test.

As shown in Table 3, the total score of perceived consequences for research misconduct increased from 35.00 (28.00,35.00) to 35.00 (35.00,35.00) in the CBL group (*P* < 0.001), and the differences in the total score were also statistically significant in the control group with a *P* value of 0.015. All the listed items showed significant score increases in the CBL group (*P* < 0.01), but “the rational allocation of research resources”, “public trust in researchers”, “the entire academic environment” and “research integrity throughout the society” only increased in the control group (*P* < 0.05).

**Table 3:** Comparison of perceived consequences for research misconduct before and after the courses [Median (IQR)].

Items	CBL group^a^ (n=149)		Control group^a^ (n=149)	
	Pre-course	Post-course	Pre-course	Post-course
Personal academic reputation	5.00(4.00,5.00)	5.00(5.00,5.00)**	5.00(4.00,5.00)	5.00(5.00,5.00)
The reputation of the institution and academic community	5.00(4.00,5.00)	5.00(5.00,5.00)*	5.00(4.00,5.00)	5.00(4.00,5.00)
The normal progression of research activities	5.00(4.00,5.00)	5.00(5.00,5.00)**	5.00(4.00,5.00)	5.00(4.00,5.00)
The rational allocation of research resources	5.00(4.00,5.00)	5.00(5.00,5.00)**	5.00(4.00,5.00)	5.00(4.00,5.00)*
Public trust in researchers	5.00(4.00,5.00)	5.00(5.00,5.00)**	5.00(4.00,5.00)	5.00(5.00,5.00)*
The entire academic environment	5.00(4.00,5.00)	5.00(5.00,5.00)**	5.00(4.00,5.00)	5.00(5.00,5.00)*
Research integrity throughout the society	5.00(4.00,5.00)	5.00(5.00,5.00)***	5.00(4.00,5.00)	5.00(5.00,5.00)*
Total score	35.00(28.00,35.00)	35.00(35.00,35.00)***	35.00(28.50,35.00)	35.00(32.00,35.00)*

^*^ *P* < 0.05.

^**^ *P* < 0.01.

^***^ *P* < 0.001.

^a^The comparison before and after the course was conducted using the Mann-Whitney U test.

Table 4 presents the comparison of agreement rates regarding research misconduct before and after the courses. The agreement rates regarding research misconduct decreased in both groups (*P* < 0.05). All the listed items showed significant decreases in the agreement rate in the CBL group (*P* < 0.05), but only “multiple submissions”, “duplicate publication”, “Providing false academic information in the process of the project application, application of achievement, award, or title, or degree application” and “Joining the authorship without participating in the research or creation, listing others as authors without their permission, listing fake names as authors, or not listing authors who made contributions to the research and manuscript” showed statistically significant decreases in the control group (*P* < 0.05).

**Table 4:** Comparison of agreement rate regarding research misconduct before and after the courses [n(%)].

Items	CBL group^a^ (n=149)		Control group^a^ (n=149)	
	Pre-course	Post-course	Pre-course	Post-course
Multiple submissions	47(31.5)	6(4.1)***	49(32.9)	9(6.0)***
Duplicate publication	25(16.8)	4(2.7)***	23(16.8)	7(4.7)**
Buying and selling papers, letting other people write papers, or writing papers for others	15(10.1)	3(2.0)**	10(6.7)	4(2.7)
Plagiarizing or misappropriating others’ academic achievements	14(9.4)	3(2.0)**	9(6.0)	5(3.4)
Falsifying research data, materials, literature or annotations, or fabricating research results	13(8.7)	3(2.0)*	9(6.0)	5(3.4)
Providing false academic information in the process of the project application, application of achievement, award, or title, or degree application	14(9.4)	3(2.0)**	14(9.4)	5(3.4)*
Joining the authorship without participating in the research or creation, listing others as authors without their permission, listing fake names as authors, or not listing authors who made contributions to the research and manuscript	14(9.4)	4(2.7)*	14(9.4)	4(2.7)*
Agreeing to conduct at least one of the listed research misconduct	55(36.9)	7(4.7)***	55(36.9)	11(7.4)***

^*^ *P* < 0.05

^**^ *P* < 0.01

^***^ *P* < 0.001

^a^The comparison before and after the course was conducted using the Chi-squared test.

## Discussion

Our study investigated the effectiveness of CBL and traditional lecture on residents’ knowledge and attitudes towards research misconduct, which has not been previously examined in this field. To enhance the reliability of results, we utilized PSM method to balance potential confounding factors that might affect their knowledge and attitudes, including residence year and educational status, and all the demographic characteristics were not significantly different between the two groups. The participants’ knowledge on research misconduct, perceived consequences for research misconduct, and their agreement rate regarding research misconduct significantly improved in the CBL and control group. However, certain aspects of their perceived consequences and agreement rate did not show significant improvement..

The scores of knowledge about research misconduct increased in the CBL and control group, and the differences between the two groups were not significant, and this may suggest that research misconduct is a crucial academic issue that can be effectively communicated and deeply understood through various teaching methods. CBL is not the only or the most effective way to enhance the understanding of research misconduct, and traditional lecture can also play an effective role in teaching this content. After all, traditional lecture is favored due to its necessity and effectiveness [13], particularly suitable for imparting core knowledge and concepts to large groups of audience [10].

In the CBL group, the scores of perceived consequences for research misconduct significantly increased in all aspects, while only four aspects improved in the control group. Compared with traditional lecture, CBL possesses some advantages. Firstly, students are more actively in their learning process in the CBL group, unlike traditional lecture where learners are usually passive and disinterested in class [14]. Secondly, CBL is effective in fostering the application and integration of knowledge, collaboration with peers, and problem-solving skills, rather than solely relying on the teacher-centered approach for content delivery [15]. Thirdly, CBL provides an avenue for feedback on case studies and enables learners to engage in discussions with field experts, but traditional lecture typically offers limited feedback on learners’ comprehension [16,17]. Moreover, CBL is regarded as fostering a deeper comprehension and advanced learning, contrasting with the superficial approach often associated with traditional lecture [18]. These advantages may enable participants to have a deeper understanding of the consequences for research misconduct during the teaching process, thereby generating a stronger warning effect.

The agreement rate for all research misconduct behaviors decreased significantly in the CBL group, whereas only the agreement rates for four research misconduct behaviors showed a decline in the control group. This may be attributed to the traditional lecture adopted, which lacked specific case analyses and thorough discussions [19]. Consequently, students’ understanding of research misconduct was not profound enough, making it difficult for them to develop a comprehensive awareness. “Buying and selling papers, letting other people write papers, or writing papers for others”, “Plagiarizing or misappropriating others’ academic achievements” and “Falsifying research data, materials, literature or annotations, or fabricating research results” are considered the most dangerous to research integrity due to their severe harm. However, traditional lecture has not shown statistically significant improvements in participants’ attitudes towards these three behaviors, indicating that solely relying on traditional lecture for moral education or behavioral norm promotion may not effectively change participants’ understanding and attitude towards these unethical practices. This result reflects the positive impact of CBL in shaping residents’ academic ethical concepts. Through the deep analysis and discussion of real-life cases, residents are able to more intuitively understand the harms of research misconduct, thereby forming a deep-seated aversion and vigilance against such behaviors. This decrease in agreement rates is conducive to creating an honest and fair academic atmosphere, which in turn promotes the healthy development of scientific research activities. Our previous research has shown that the higher the score of perceived consequences for research misconduct, the lower the occurrence rate of such behavior, which basically corroborates the current research findings [5].

The Kirkpatrick model is an important step in measuring the effectiveness of training, including level 1 (REACTION), level 2 (LEARNING), level 3 (BEHAVIOR) and level 4 (RESULTS) [20], which is widely used in many fields [21,22]. In this study, we observed significant improvements in the CBL group in terms of knowledge about research misconduct, perceived consequences of research misconduct, and agreement rates regarding research misconduct, indicating that the training was effective in raising participants’ awareness and attitudes towards research misconduct at the second level of Kirkpatrick model. Although it does not directly measure behavioral change, our results provide a foundation for further behavioral change. Specifically, the CBL group showed a significant increase in scores for perceiving the consequences of research misconduct, which may enhance participants’ understanding of the consequences of misconduct and thus potentially influence their behavior. Furthermore, the CBL group showed a significant decrease in agreement rates for all types of research misconduct, indicating that CBL may help participants more intuitively understand the harm of research misconduct through in-depth analysis and discussion of real-life cases, thereby forming a deep-seated aversion and vigilance against such behaviors, which may help reduce the occurrence of these behaviors. To more directly assess the impact of training on behavior, future research could consider the third and fourth of Kirkpatrick model by tracking participants’ actual behavioral changes after training to evaluate the effectiveness of the training.

Although our study provided valuable insights into the effectiveness of CBL compared to traditional lecture in shaping residents’ knowledge and attitudes towards research misconduct, there are some limitations that need to be acknowledged. Firstly, due to the potential subjective influence of researchers on the selection of training content and cases, as well as the presentation style, there may introduce some biases. Secondly, this study did not conduct long-term follow-up surveys to verify the effectiveness of the training, making it impossible to determine whether the training truly had a positive impact on the participants’ behavior. Additionally, the CBL and traditional lecture groups included residents from different hospitals, and it is inevitable that there are differences in residents’ scientific literacy despite of the use of PSM method, which may influence the generalizability of our findings. Lastly, our study focused primarily on residents, and the results might not be directly applicable to other populations or professional groups. Future research could extend the investigation to a broader range of participants to assess the generalizability of our findings.

## Conclusions

In summary, our study explored the impact of CBL and traditional lecture on residents’ comprehension and perspectives towards research misconduct, an area that had not been thoroughly investigated before. The results revealed that in the CBL group, there is a marked enhancement in residents’ knowledge about research misconduct, their perception of its consequences, and their overall disapproval of such behavior. This underscores the effectiveness of CBL in fostering a deeper understanding and stronger aversion towards research misconduct among residents.
